# Amplification of enantiomeric excess by dynamic inversion of enantiomers in deracemization of Au_38_ clusters

**DOI:** 10.1038/s41467-020-18357-0

**Published:** 2020-09-11

**Authors:** Yanan Wang, Belén Nieto-Ortega, Thomas Bürgi

**Affiliations:** Department of Physical Chemistry, University of Geneva, 1211 Geneva, Switzerland

**Keywords:** Chemistry, Materials science, Nanoscience and technology

## Abstract

Symmetry breaking and amplification processes have likely played a fundamental role in the development of homochirality on earth. Such processes have not been much studied for inorganic matter at the nanoscale. Here, we show that the balance between left- and right-handed intrinsically chiral metal clusters can be broken by adsorbing a small amount of a chiral molecule in its ligand shell. We studied the amplification of enantiomeric excess of the Au_38_(2-PET)_24_ cluster (2-PET = 2-phenylethylthiolate). By exchanging a small fraction of the achiral 2-PET ligand by chiral R-1,1′-binaphthyl-2,2′-dithiol (R-BINAS), a mixture of species is obtained composed of anticlockwise (A) and clockwise (C) versions of Au_38_(2-PET)_24_ and Au_38_(2-PET)_22_(R-BINAS)_1_. At 70 °C, the system evolves towards the anticlockwise clusters at the expense of the clockwise antipode. It is shown that the interplay between the diastereospecific ligand exchange, which introduces selectivity but does not change the A/C ratio, and the fast racemization of the Au_38_(2-PET)_24_ is at the origin of this observation.

## Abstract (web summary)

Symmetry breaking and amplification processes play a fundamental role in nature and technology. Here, the authors show that the interplay between racemization and ligand exchange leads to amplification of enantiomeric excess of intrinsically chiral metal clusters.

## Introduction

Chirality, which describes the symmetry properties of matter, is ubiquitous in nature^1,2^ at very different length scales ranging from the very big down to the size of molecules. Many molecules of life, such as glucose, amino acid, and DNA, possess handedness, with tremendous consequences for example for the pharmaceutical industry, because enantiomers of a chiral molecule behave differently in a chiral environment. The development of chiral drugs^3^ and catalysts^4^, which afford high enantioselectivity therefore remains a central aspect of current chemical sciences. In addition, in the field of nanoscale materials, chirality significantly extends their application potential in optics (metamaterials) and materials science^5–8^. Chiral nanomaterials have therefore shifted in the centre of interest of many research groups^9–11^.

Thiolate-protected gold nanoclusters Au_m_(SR)_n_, a special class of ultrasmall, atomically precise nanomaterials, have molecule-like and size-dependent properties, which make them highly attractive for applications in fields like biology or catalysis, where chirality plays a central role. The preparation of chiral nanoclusters thus became an unremitting ambition in this field. Chirality in these systems can be imparted at different levels. Considering the general formula Au_m_(SR)_n_ chirality can be due to the ligands SR. Such chiral clusters can be prepared by direct synthesis using a chiral thiol^12^. Alternatively ligand exchange is a good method to incorporate chirality to achiral clusters. For example, ligand exchange on achiral Au_25_(SR)_18_ with chiral thiols (SR*), resulted in a series of chiral Au_25_(SR)_m_(SR*)_n_ clusters with chiroptical activity^13–16^. Alternatively, Au_m_(SR)_n_ can be intrinsically chiral due to a chiral metal core or due to the chiral arrangement of thiolates on the cluster surface. Of course, combinations of the different possibilities mentioned above are also possible, e.g. chiral ligand and chiral arrangement of the ligand. The Au_38_(GS)_24_ is an example of such a cluster (GS = glutathionate)^17^. In Au_38_ the ligands are chirally arranged on the surface of a symmetric gold core. Au_38_(SR)_24_ has six dimeric (–SR–Au–SR–Au–SR–) and three monomeric staples (–SR–Au–SR–). The six dimeric staples are arranged in two propeller-like structures at the poles of the cluster. The two enantiomers of the cluster are described as A-Au_38_(SR)_24_ (anticlockwise, left-handed) and C-Au_38_(SR)_24_ (clockwise, right-handed).

The chirality of the different structural elements (core, ligand arrangement, and ligand) may not be independent of each other. In fact, it has been shown that the chirality of the Au_38_ cluster can be transferred to its ligands^18^. Specifically, the enantiomers of Au_38_(2-PET)_24_ (2-PET = 2-phenylethylthiolate) showed strong vibrational circular dichroism (VCD) signals in the 2-PET vibrations. 2-Phenylethylthiol is an achiral molecule without VCD activity. However, due to the interaction with the cluster, 2-PET adopts a chiral gauche conformation with preferential handedness. Put in other words: the cluster imposes its chirality onto the ligand. We reasoned whether the inverse could be possible as well namely that a chiral ligand imposes its chirality onto the cluster therefore leading to a deracemization of the cluster framework.

Deracemization^19,20^ is the transformation of a racemic mixture into a nonracemic mixture by increasing the quantity of one enantiomer at the expense of the other. Due to the importance of enantiopure compounds in pharmaceutical industry and catalysis, in the past decades various deracemizational strategies were developed^19^ and major works concentrated on using chemical reagents to separate or transform enantiomers^19,21–23^. In addition, recently many emerging technologies have also been developed to achieve this goal. Crystallisation of a racemic mixture is an effective approach to enrich one enantiomer by the formation of conglomerate crystals^24,25^ or through spontaneous deracemization during crystallisation^26,27^. Futhermore, coherent laser light^28^ and highly enantioselective enzymes^29^ have been used for efficient deracemization. The development of deracemization strategies has brought tremendous impetus to the pharmaceutical and catalysis fields^3,19,30^. By contrast, the deracemization of gold nanoclusters, especially intrinsic chiral nanoclusters, has not been reported up to now.

The Au_38_ cluster seems an ideal candidate for such a study. The cluster enantiomers can be easily separated using chromatography^31^ and the cluster racemizes at reasonably low temperature^32^, which is also the basis for a deracemization imposed by the presence of a chiral ligand.

**Fig. 1:**
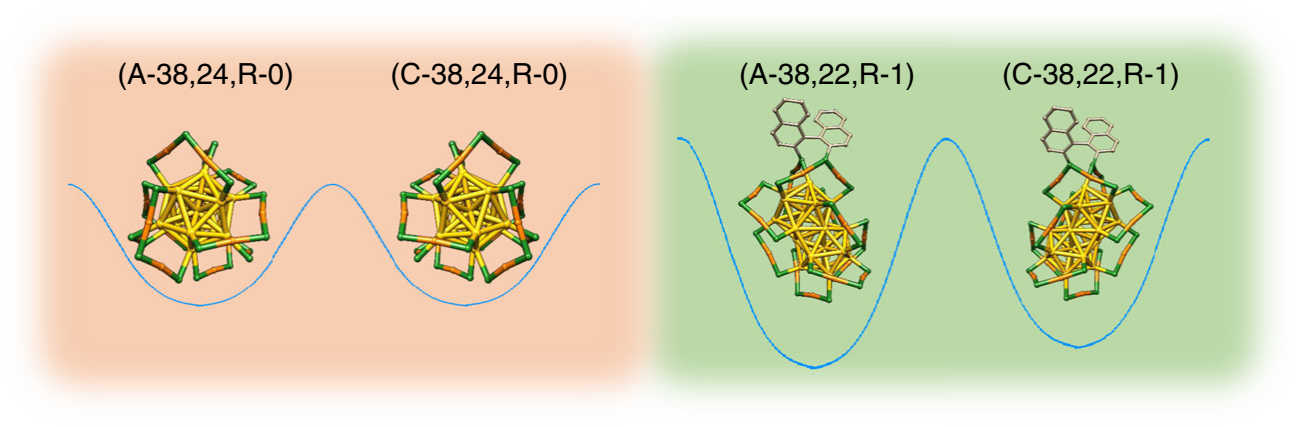
Potential energy curves of Au_38_(2-PET)_24_ enantiomers and R-BINAS-substituted diastereomers. Color code: yellow, Au_core_; orange, Au_adatom_; green, S. The 2-PET ligand is omitted for clarity. Nomenclature: (A-38, 22, R-1) means anticlockwise cluster with one R-BINAS and 22 2-PET ligands in its ligand shell.

The activation barrier for the racemization of Au_38_, 28.14 ± 0.53 kcal mol^−1^, is lower than the energy of a gold-sulfur bond^32^. This means that racemization takes place without complete Au–S bond breaking^32^. Very recently Häkkinen and coworkers proposed a mechanism for this inversion of the Au–S framework of Au_38_(2-PET)_24_ (ref. ^33^). In this model no Au–S bonds are broken and the racemization proceeds via a rotational reconstruction of the metal core. Interestingly, after introducing a rigid di-thiolate, 1,1′-binaphthyl-2,2′-dithiol (BINAS), into the ligand shell of the cluster the racemization drastically slows down^34^. For example at 70 °C the racemization of Au_38_(2-PET)_22_(BINAS)_1_ is about 27 times slower compared to the parent cluster. Schematic potential energy diagrams are illustrated in Fig. 1, where A and C (anticlockwise, clockwise) describe the absolute configuration of the cluster Au–S framework.

The inversion of the cluster is, however, not the only dynamic process of the system to be considered. In fact, thiolate-protected clusters can exchange ligands among each other upon collision^35^, which has been shown for monothiol ligands. Here we further demonstrate such ligand exchange among clusters for a dithiol ligand. In the following we show that indeed the chirality of a thiolate-protected gold cluster can be amplified by the presence of a chiral thiol in its ligand shell. The phenomenon relies on the dynamic nature of the clusters and we show that diastereospecific ligand exchange between clusters (in absence of free ligand) plays a crucial role.

## Results

### Characterization and racemization of Au_38_ clusters

*Rac*-Au_38_(2-PET)_24_ (racemic) clusters were synthesized and purified as reported^31^. The MALDI and UV-vis data of *rac*-Au_38_(2-PET)_24_ are shown in Supplementary Fig. 1. In chiral HPLC the sample showed two well-separated peaks with retention times of 38 min and 81 min corresponding to the two enantiomers of the Au_38_ cluster. The absolute configuration of the two enantiomers was successfully attributed using CD spectroscopy by comparison with calculated spectra^31^. The first (second) peak corresponds to the anticlockwise (clockwise) form of Au_38_(2-PET)_24_. R-BINAS was introduced via ligand exchange by mixing *rac*-Au_38_(2-PET)_24_ and R-BINAS at molar ratio 1:100. The extent of ligand exchange depends on the reaction time. After some time the reaction mixture was passed over a size-exclusion column to remove the free ligand. An example of the MALDI and HPLC data of the reaction mixture is shown in Supplementary Fig. 2. In addition to the first two peaks, which belong to the enantiomers of the Au_38_(2-PET)_24_ cluster, several other peaks were observed in HPLC. Based on previous MALDI^34,36^ (composition) and CD analysis (absolute configuration of the metal cluster)^31^, those peaks in the chromatogram were assigned to different species containing R-BINAS in their ligand shell. We will use the following nomenclature to describe these species: The anticlockwise cluster with one R-BINAS and 22 2-PET ligands in its ligand shell will be called (A-38, 22, R-1). Peaks at 107, 152, and 270 min, are assigned to (A-38, 22, R-1), (C-38, 22, R-1), and (A-38, 20, R-2), respectively. We should mention that here R-BINAS was chosen as the chiral ligand because with S-BINAS the separation of the resulting cluster species in the chromatograms is not as good (Supplementary Fig. 2), which complicates quantitative analysis. We note that the ligand exchange was found to be diastereoselective^37^. Specifically, R-BINAS reacted with anticlockwise cluster (A-38, 24, R-0) four times faster than with clockwise cluster (C-38, 24, R-0), as has been shown by analyzing the kinetics of the reaction. Therefore (A-38, 22, R-1) is more abundant than (C-38, 22, R-1) and (C-38, 24, R-0) is more abundant than (A-38, 24, R-0) after ligand exchange reaction^37^. This is directly evident from the HPLC data (Supplementary Fig. 2) of a sample after ligand exchange. The areas under the peaks reflects the relative abundances of the corresponding specises. For example, the ratio of (A-38, 22, R-1) to (C-38, 22, R-1) is around 3.1 derived from the integration of the two peaks at 107 and 152 min. However, ligand exchange does not affect the relative abundance of clockwise and anticlockwise clusters in the system. The latter quantity can only be affected by an inversion of the Au–S framework.

Racemization is not the only process to be considered, as mentioned above. It has been shown that ligands can exchange between clusters even if no free ligands are present^35^. We therefore verified if R-BINAS can exchange between clusters. Experiments with Au_25_(2-PET)_18_ and Au_38_(2-PET)_24-2x_(R-BINAS)_x_ (average number of x = 2) were performed. The mixture of Au_25_ and Au_38_ was heated to 70 °C for 24 h followed by analysis by MALDI-TOF (Supplementary Fig. 3). Ligand exchange between the clusters was clearly evidenced by the observation of Au_25_(2-PET)_16_(R-BINAS)_1_. Therefore ligand exchange between clusters has to be considered in the following discussion.

### Deracemization of Au_38_ clusters

**Fig. 2:**
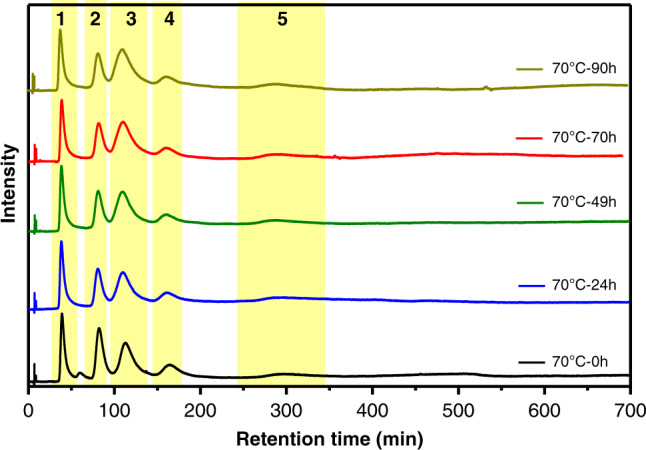
Exemplary HPL chromatograms for deracemization experiment. The sample was prepared by ligand exchange of *rac*-Au_38_(2-PET)_24_ with R-BINAS, followed by removal of free ligands. On average the number of incorporated R-BINAS in this sample was $\overline x _{R - {\mathrm{BINAS}}} = 0.514$, as determined from the HPL chromatograms. The sample was then heated to 70 °C and chromatograms were measured as a function of time. The peaks are assigned as follows: Peak **1**, (A-38, 24, R-0); Peak **2**, (C-38, 24, R-0); Peak **3**, (A-38, 22, R-1); Peak **4**, (C-38, 22, R-1); Peak **5**, (A-38, 20, R-2).

For the deracemization studies, the sample after ligand exchange reaction and after removing any free ligand was heated to 70 °C in toluene, and HPL chromatograms were recorded at different reaction times (up to 4 days) as illustrated in Fig. 2 in order to follow the evolution of the different species. 70 °C was chosen because at this temperature racemization of the Au_38_(2-PET)_24_ cluster is fast whereas inversion of the Au–S framework of Au_38_(2-PET)_22_(R-BINAS)_1_ is slow. We note that the anticlockwise (A) and clockwise (C) versions of the latter clusters are not true enantiomers but diastereomers; however, their Au–S framework have opposite absolute configuration. From Fig. 2 it is evident that the relative peak areas evolve with time. This is may be most evident from the comparison of peaks 2 and 3, which change relative intensity. Most importantly, the relative intensity of peaks 3 and 4, belonging to (A/C-38, 22, R-1) species showed sign of a deracemization of the Au–S cluster framework. At the beginning of the experiment the anticlockwise cluster (A-38, 22, R-1) was more abundant due to the diastereoselective ligand exchange during sample preparation. However, in the course of the experiment, in absence of free ligand, the abundance of this cluster with respect to its antipode (C-38, 22, R-1) further increased. The behavior of the unexchanged A/C-Au_38_(2-PET)_24_ cluster is different. Due to the diastereoselective ligand exchange^37^, the clockwise version is more abundant at the beginning of the experiment. As expected, result from the fast racemization at 70 °C, the relative abundance of the enantiomers approached a racemic mixture. Most importantly, the total abundance of anticlockwise clusters in the system ((A-38, 24, R-0) + (A-38, 22, R-1)) increased with respect to the clockwise clusters ((C-38, 24, R-0) + (C-38, 22, R-1)). This ratio will be called A/C ratio in the following.

**Fig. 3:**
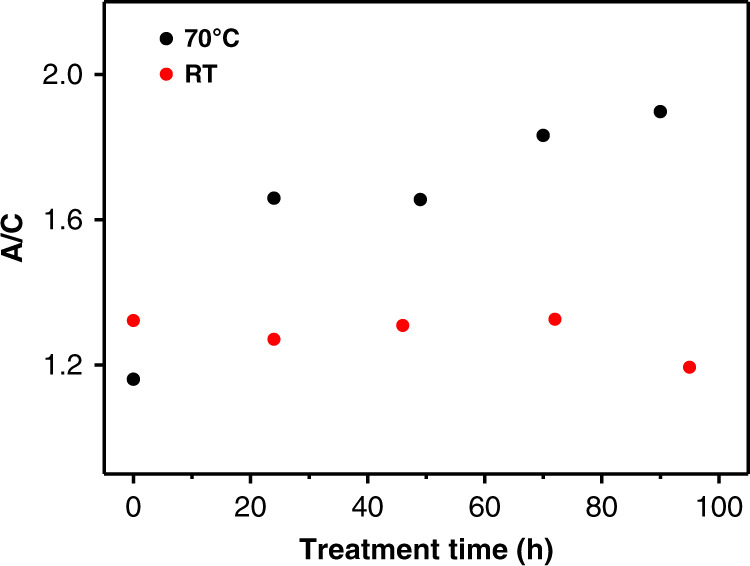
Ratio of anticlockwise (A) to clockwise (C) clusters as a function of time. Experiments were performed at room temperature (red points, $\overline x _{R - {\mathrm{BINAS}}} = 0.526$) and at 70 °C (black points, $\overline x _{R - {\mathrm{BINAS}}} = 0.514$). Concentrations of cluster species were derived from peak areas in HPL chromatograms. Anticlockwise clusters (A): (A-38, 24, R-0)+(A-38, 22, R-1), clockwise clusters (C): (C-38, 24, R-0)+(C-38, 22, R-1).

The increase of the anticlockwise (A) to clockwise (C) ratio of the clusters as a function of time is shown in Fig. 3. The black points correspond to the experiments performed at 70 °C (data extracted from chromatograms shown in Fig. 2). The ratio increased from initially 1.16 to 1.90. For comparison the corresponding data is also shown for an experiment performed at room temperature (red points), for which the chromatograms are shown in Supplementary Fig. 4. The chromatograms exhibited no obvious changes in this case, which is also reflected in Fig. 3. This shows that the process can be initiated by adjusting the temperature. Even for lower average number of incorporated R-BINAS in Au_38_ clusters the inversion can be detected following heating, as shown in Fig. S5, where $\overline x _{R - {\mathrm{BINAS}}} = 0.225$. In this case, UV-vis spectra of the Au_38_(2-PET)_24-2x_(R-BINAS)_x_ cluster mixture were measured after different heating times as shown in Supplementary Fig. 6. Even after extended heating the UV-vis spectra exhibit the typical features of Au_38_(2-PET)_24_. This means the cluster is stable under these conditions and no significant decomposition was observed, in agreement with HPLC data, which do not give evidence for additional species formed. From the UV-vis and CD spectra the concentration-independent anisotropy factors g = θ[mdeg] (32,980 × A)^−1^ were calculated (Supplementary Fig. 7). With longer heating time, the anisotropy factor of the sample increased. As the amount of R-BINAS was constant throughout the experiment, the increased chiroptical signals are derived from the combination of (1) the inversing of the Au–S cluster framework and (2) the changing distribution of R-BINAS on the two enantiomers in combination with the changes in the CD spectra induced by the presence of R-BINAS on the two enantiomers of the cluster. The chiroptical signal originating from the Au–S framework is expected to be more intense compared with the changes induced by the chiral ligand. However, the latter contribution to the optical activity hinders quantitative analysis of the CD data^38^.

### BINAS content dependence of deracemization of Au_38_ clusters

**Fig. 4:**
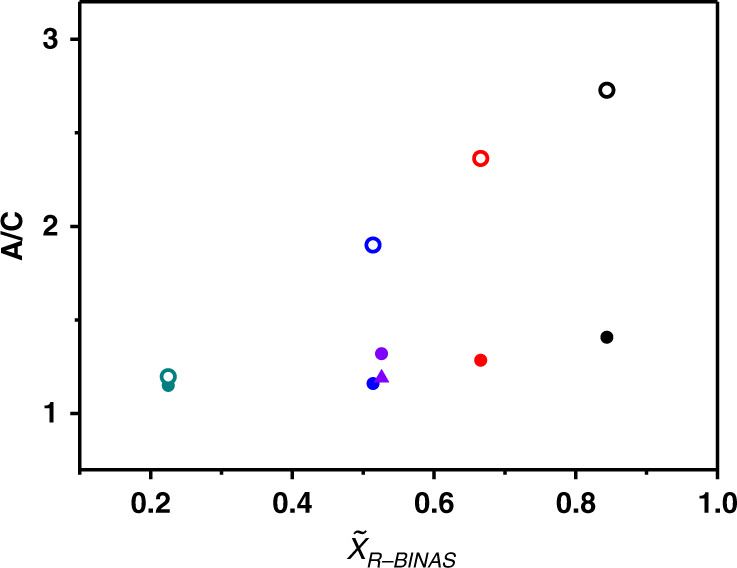
Dependence of A/C ratio on the average number of incorporated BINAS ligands $\overline x _{R - {\mathrm{BINAS}}}$. Solid spots, before thermal treatment; hollow spots, after thermal treatment at 70 °C for more than 70 h. Color code: green, $\overline x _{R - {\mathrm{BINAS}}} = 0.225$; blue, $\overline x _{R - {\mathrm{BINAS}}} = 0.514$; red, $\overline x _{R - {\mathrm{BINAS}}} = 0.666$; black, $\overline x _{R - {\mathrm{BINAS}}} = 0.844$; violet, $\overline x _{R - {\mathrm{BINAS}}} = 0.526$ (triangle spot: sample kept at room temperature for 95 h).

A key parameter affecting the deracemization is the average number of R-BINAS ligands $\overline x _{R - {\mathrm{BINAS}}}$ in the clusters, which was controlled by the ligand exchange time during the preparation of the sample. This number was determined from integrated areas of the first four peaks in the HPL chromatograms (species with x = 0 and x = 1). Au_38_ cluster samples with different average number of R-BINAS in their ligand shell where then used as the starting material for deracemization experiments at 70 °C. The time evolution of the HPL chromatograms for these experiments are shown in Supplementary Figs. 5, 8, 9. At very low R-BINAS content of $\overline x _{R - {\mathrm{BINAS}}} = 0.225$ (Supplementary Fig. 5) the changes in the relative peak areas after heating at 70 °C up to 90 h were very small. At higher R-BINAS content, for instance $\overline x _{R - {\mathrm{BINAS}}} = 0.666$ (see Supplementary Fig. 8), obvious changes of the abundance of different cluster species during thermal treatment could be observed. Figure 4 summarizes these experiments by showing the A/C ratio at the beginning of the experiment and after thermal treatment as a function of R-BINAS content $\overline x _{R - {\mathrm{BINAS}}}$. From this plot it is clear that, irrespective of the R-BINAS content, the amount of anticlockwise clusters increased during thermal treatment. Furthermore, the clusters with higher $\overline x _{R - {\mathrm{BINAS}}}$ expressed larger increases of the A/C ratio, which means a higher tendency of the Au–S framework to invert to the anticlockwise form. Specially, for the experiment with the highest R-BINAS content ($\overline x _{R - {\mathrm{BINAS}}} = 0.844$, Supplementary Fig. 9) the final A/C ratio was 2.7 corresponding to an enantiomeric excess of 46%. This result illustrates that the driving force for the deracemization is related to the abundance of R-BINAS in the ligand shell of the Au_38_ cluster.

The experiments reveal an increase of (A-38, 22, R-1) even when this cluster is already in excess at the beginning of the experiment. These observations may partly be due to the inversion of (A/C-38, 22, R-1). However, ligand exchange reactions between clusters couples the two cluster populations (A/C-38, 24, R-0) and (A/C-38, 22, R-1), thus they are not independent from each other. A strong indication for this is the observation that the cluster without R-BINAS (A/C-38, 24, R-0) does not reach the racemic state even after long heating at 70 °C. This is most evident from the experiments with high R-BINAS content (see for example Supplementary Fig. 9, $\overline x _{R - {\mathrm{BINAS}}} = 0.844$).

### Kinetic model of the dynamic system

**Fig. 5:**
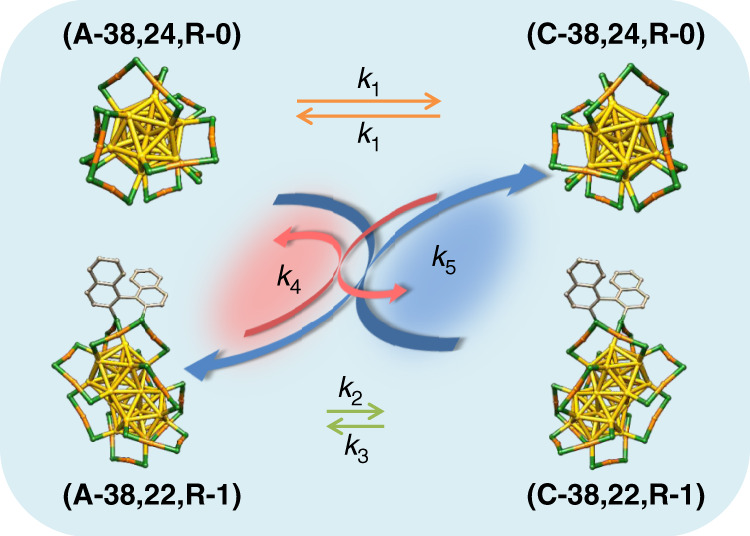
Schematics of the reactions, which are considedred in the dynamic system. The symbols *k*_*1*_ to *k*_*5*_ stand for the rate constant of the corresponding reaction.

In order to better understand the bahaviour of this dynamic system we simulated its kinetics using a model (see MATLAB code in the Supplementary note 1). As elementary reactions we considered (1) the racemization of the Au_38_(2-PET)_24_ cluster, (2) the racemization of the Au–S framework of the Au_38_(2-PET)_22_(R-BINAS)_1_ cluster and (3) the ligand exchange between clusters. Explicit chemical equations for the three reactions are given below and are schematically illustrated in Fig. 5:

1 $$A - {\mathrm{Au}}_{38}\left( {2 - {\mathrm{PET}}} \right)_{24}\begin{array}{*{20}{c}} {\mathop { \to }\limits^{k_1} } \\ {\mathop { \leftarrow }\limits_{k_1} } \end{array}C - {\mathrm{Au}}_{38}\left( {2 - {\mathrm{PET}}} \right)_{24}$$

2 $$A - {\mathrm{Au}}_{38}\left( {2 - {\mathrm{PET}}} \right)_{22}\left( {R - {\mathrm{BINAS}}} \right)_1 \\ \begin{array}{*{20}{c}} {\mathop { \to }\limits^{k_2} } \\ {\mathop { \leftarrow }\limits_{k_3} } \end{array}C - {\mathrm{Au}}_{38}\left( {2 - {\mathrm{PET}}} \right)_{22}\left( {R - {\mathrm{BINAS}}} \right)_1$$

3 $$A - {\mathrm{Au}}_{38}\left( {2 - {\mathrm{PET}}} \right)_{22}\left( {R - {\mathrm{BINAS}}} \right)_1 \,+\, C - {\mathrm{Au}}_{38}\left( {2 - {\mathrm{PET}}} \right)_{24}\\ \begin{array}{*{20}{c}} {\mathop { \to }\limits^{k_4} } \\ {\mathop { \leftarrow }\limits_{k_5} } \end{array}C - {\mathrm{Au}}_{38}\left( {2 - {\mathrm{PET}}} \right)_{22}\left( {R - {\mathrm{BINAS}}} \right)_1 \,+\, A - {\mathrm{Au}}_{38}\left( {2 - {\mathrm{PET}}} \right)_{24}$$

From independent experiments^32,34^ we know that *k*_*1*_ is 27 times faster than *k*_*2*_. We furthermore note that processes in Eq. (3), ligand exchange between clusters, can be obtained by combining processes in Eq. (1) and Eq. (2), which means the five rate constants are not independent. Concretely, the ratio *k*_*2*_/*k*_*3*_ is equal to *k*_*4*_/*k*_*5*_ . Importantly, the intercluster ligand exchange reactions (Eq. (3)) do not change the global A/C ratio.

We used the data of the experiment with $\overline x _{R - {\mathrm{BINAS}}} = 0.666$ (Supplementary Fig. 8) as a reference for the modeling. The time-dependent concentrations of the species in the experiment is shown in Supplementary Fig. 10. Note that the concentrations were referenced to the concentration of (A-38, 24, R-0), which was set to 1 at the beginning. Modeling was then done by taking into account the relative concentrations of the four clusters (A-38, 24, R-0), (C-38, 24, R-0), (A-38, 22, R-1), and (C-38, 22, R-1) at the beginning of the experiment as determined from the peak areas in the corresponding chromatograms as [1.0, 1.60, 3.37, and 1.80]. The rate constants *k*_*4*_ and *k*_*5*_ were adjusted in order to reproduce the experimental data, whereas fixing *k*_*1*_ and *k*_*2*_ at the ratios given above. When setting *k*_*2*_ = *k*_*3*_ and *k*_*4*_ = *k*_*5*_, modeling was not able to reproduce the increase of the concentration of (A-38, 22, R-1) but readily produced racemic mixture. When setting *k*_*2*_ > *k*_*3*_ and *k*_*4*_ > *k*_*5*_, modeling produce the increase of the concentration of (C-38, 22, R-1) which is opposite to our observation. Only by setting *k*_*2*_ < *k*_*3*_ and *k*_*4*_ < *k*_*5*_, the increase of (A-38, 22, R-1) at the expense of (C-38, 22, R-1) could be modeled (Supplementary Fig. 11). This means that the ligand exchange between clusters is diastereoselective, which is a critical point in this system. As mentioned above the ligand exchange does not alter the anticlockwise/clockwise (A/C) ratio of the clusters in the system. However, we note that the reaction associated with rate constant *k*_*5*_ produces (C-38, 24, R-0), which easily inverts to (A-38, 24, R-0) at 70 °C and hence the combination of the diastereoselective ligand exchange and the fast inversion (racemization) of the (C-38, 24, R-0) cluster leads to the overall increase of the A/C ratio.

The reaction scheme considered here bears similarities to some known processes. For instance it is similar to a dynamic kinetic resolution^39–41^, in which two enantiomers, which interconvert fast, react at different rates to form the corresponding products. In contrast to a conventional dynamic kinetic resolution, in our case the reactants (A/C-38, 24, R-0) react with the products of the other enantiomer (C/A-38, 22, R-1) in the diastereoselective process. Furthermore, this dynamic process is also similar to Viedma ripening, which is a solid-state method for the deracemization of racemic mixtures of crystalline compounds into single chirality, simply by continuously grinding a suspension^42–44^. Viedma ripening has been used in deracemization of a varity of chiral compounds but not in the cluster field.

In order to gain further confidence in our modeling we then fixed the rate constants found to reproduce the time evolution of the cluster species in the experiment shown in Supplementary Fig. 8 and changed the initial concentrations in order to verify if the model correctly reproduces the experimentally observed increase (Fig. 4) of the ovrall A/C ratio with increasing R-BINAS content. The comparison of the final A/C ratios of the experiment and the simulations agree well (results in Supplementary Fig. 12) and the simulations are able to reproduce the increase of the A/C ratio with increasing R-BINAS content. Our simulations indicate a ratio *k*_*4*_/*k*_*5*_ (= *k*_*2*_/*k*_*3*_) of 0.24.

It is worth to mention here that at elevated R-BINAS content there is evidence of doubly exchanged species (A-38, 20, R-2), peak **5** in the chromatograms. We did not try to include these species in the modeling as the number of reactions to consider would drastically increase. Instead we tried to keep the concentration of these species low by choosing low average R-BINAS content ($\overline x _{R - {\mathrm{BINAS}}}$). Some of the discrepancies between experiment and simulations (e.g., in Supplementary Fig. 12) could therefore arise from neglecting higher exchanged species in the modeling. We also note that the initial overall A/C ratio is not exactly 1 at the beginnig of the experiments and slightly increases with $\overline x _{R - {\mathrm{BINAS}}}$. The exact reason for that is unknown, probably the processes described above take place slowly already during the preparation of the starting sample by ligand exchange.

We furthermore simulated a system that initially contained equal amounts of clockwise and anticlockwise clusters. We used the same R-BINAS content as in the experiment shown in Supplementary Fig. 10 (simulation shown in Supplementary Fig. 11). As shown in Supplementary Fig. 13 the system deracemizes to basically the same final ratio A/C (A/C = 2.395 in Supplementary Fig. 11, and A/C = 2.394 in Supplementary Fig. 13). This shows that the important factor is the R-BINAS content in the system but not the initial A/C ratio.

**Fig. 6:**
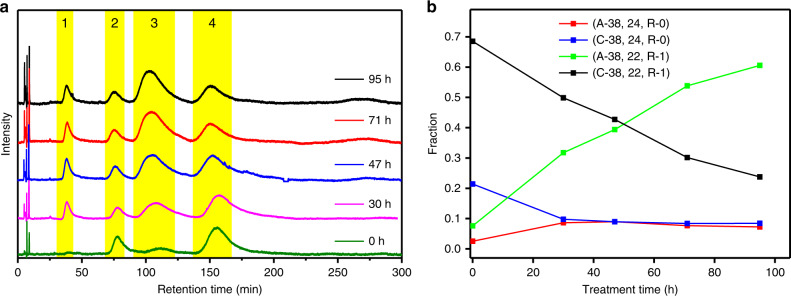
Experiment showing the conversion of clockwise to anticlockwise clusters. HPL chromatograms (**a**) and evolution of different clusters species as a function of time (**b**). The sample was prepared by ligand exchange reaction of (C-38, 24, R-0) and R-BINAS. Some enantiopure cluster (C-38, 24, R-0), was added to adjust the average number of incorporated R-BINAS in the sample, which was $\overline x _{R - {\mathrm{BINAS}}} = 0.76$. The sample was then heated to 70 °C and chromatograms were measured as a function of time. The peaks are assigned as follows: Peak **1**, (A-38, 24, R-0); Peak **2**, (C-38, 24, R-0); Peak **3**, (A-38, 22, R-1); Peak **4**, (C-38, 22, R-1).

To further confirm the transformation of clockwise to anticlockwise clusters, a sample was prepared that contained initially a high fraction of clockwise clusters (with $\overline x _{R - {\mathrm{BINAS}}} = 0.76$). This sample was prepared by isolating (C-38, 22, R-1) and (C-38, 24, R-0) clusters by semi-preparative HPLC. As the separation was not perfect some anticlockwise clusters were also present and the initial A/C ratio was 0.11. The sample was then heated to 70 °C and HPL chromatograms were measured as a function of time (Fig. 6a). The (A/C-38, 24, R-0) clusters reached a nearly racemic state quite rapidly, as expected. By contrast, the (A/C-38, 22, R-1) clusters changed from predominantly clockwise to predominantly anticlockwise. After 95 h heating, the A/C ratio increased to 2.11 (from 0.11) and continued increasing. The experiment clearly shows the predominance of anticlockwise clusters in this dynamic system (Fig. 6b).

In the dynamic situation described here, the interplay between diastereoselective intercluster ligand exchange and fast racemization of (A/C-38, 24, R-0) leads to amplification of the enantiomeric excess of the cluster (Au–S framework). This shows that the dynamic nature of these thiolate-protected gold clusters enables them to respond to changes in their environment (in the current example a chiral ligand) by shifting equilibria. This property may be of use for future applications for example, as shown here, to amplify enantiomeric excess.

In summary, we present the first successful deracemization of a thiolate-protected nanocluster. Mixtures of Au_38_(2-PET)_24_ and Au_38_(2-PET)_22_R-BINAS were prepared by ligand exchange reactions. The concentration of the four species in the system evolved with time at 70 °C, leading to an accumulation of the anticlockwise version of the Au_38_(2-PET)_22_R-BINAS cluster, at the expense of the clockwise antipode. The overall excess of the anticlockwise clusters in the system increased with increasing R-BINAS content in the system. Simulations of this dynamic process revealed that ligand exchange between clusters is diastereospecific and the resulting amplification of enantiomeric excess relies on the interplay between the ligand exchange and the racemization of the cluster. This means that the selectivity step (ligand exchange) and the step, which leads to the accumulation of anticlockwise clusters (racemization), are separated. In general, our findings show that the interplay between different dynamic processes (racemization and ligand exchange), can lead to amplification phenomena at the nanoscale.

## Methods

### Chemicals

All chemicals were purchased from commercial suppliers and used as received without any further treatment. R-BINAS was synthesized from BINOL as reported before^37,45^.

### Synthesis and size-selection of *rac*-Au_38_(2-PET)_24_

One gram of HAuCl_4_∙3H_2_O and 3.12 g of L-glutathione were dissolved in 100 mL of acetone in a 500 mL round bottom flask, and stirred vigorously at room temperature for 5–10 min. Then the flask was put into an ice bath and kept at 0 °C for 20–25 min, obtaining a yellow suspension. Freshly prepared NaBH_4_ (960 mg) in 30 mL H_2_O (0 °C) was added to the solution, which turned from yellow–white to black–brown immediately. This solution was kept stirring for 20 min at 0 °C at lower stirring speed. After that, the black precipitate was stuck on the wall of the flask. Acetone was removed and the precipitate was dried with air. The clusters were redissolved in a mixture of H_2_O (30 mL), EtOH (1.6 mL), and toluene (10 mL), followed by addition of 10 mL of 2-phenylethanethiol to the clusters. The system was heated to 80 °C and reacted for 24 h. After cooling to room temperature, large amounts of methanol were added to the flask and left overnight. Methanol was removed by decanting and filtration. The collected clusters were rinsed with methanol, dissolved in DCM and dried by rotatory evaporation. This washing step was repeated several times to remove free thiol and small clusters. Size-exclusion chromatography (SEC column) was used to purify the raw clusters. Purity of the Au_38_(2-PET)_24_ sample was verified by UV-vis spectroscopy, MALDI-TOF mass spectrometry and HPLC.

### Ligand exchange reactions

Purified rac-Au_38_(2-PET)_24_ and enantiopure R-BINAS were dissolved in toluene (1 mg mL^−1^) at a molar ratio of clusters to BINAS of 1:100. Ligand exchange was performed at room temperature. After reaction, the solution was concentrated and immediately passed over a size-exclusion column to remove the free ligands. The collected product was then ready for use in the HPLC experiments.

### HPL chromatography

Chiral HPLC was performed on a JASCO 20XX HPLC system equipped with a semi-preparative Phenomenex Lux-5u-cellulose-1 column (5 μm, 250 × 10 mm). The sample was injected in toluene and eluted with n-hexane/iso-propanol 75:25 at a flow rate of 2.5 mL/min. A JASCO 2070 plus UV-vis detector was used for the detection. For thermal treatment, the concentrated sample was heated in an oil bath to 70 °C and injected to HPLC after various times. Peak areas were determined using PeakFit (seasolve, version 4.12). Fitting was performed after subtracting a background and by using chromatography peak type (exponentially modified Gaussian) with varying peak width and shape. Whereas the standard error for the fitted areas was usually on the order of 1–2% it could be somewhat larger in cases where the background of the chromatogram was not well behaved. Consequently, the standard error for the A/C ratio was typically smaller than 4%.

### UV-vis and CD spectroscopy

UV-vis spectra were measured on a Varian Cary 50 spectrometer. A quartz cuvette of 2 mm path length was used. CD spectra were recorded at the same conditions on a JASCO J-815 CD-spectrometer. For each CD spectrum ten scans were averaged at a scanning speed of 200 nm min^−1^ with a data pitch of 1 nm. The anisotropy factors g = θ[mdeg] (32,980 × A)^−1^ was calculated from the UV-vis and CD spectra^38^.

### MALDI-TOF-MS

Mass spectra were recorded on a Bruker Autoflex mass spectrometer in positive linear mode with a nitrogen laser at near-threshold laser intensity. As matrix *trans-2-[3-(4-tert-Butylphenyl)-2-methyl-2-propenylidene]-malononitrile* was used. 3.5 mg matrix was dissolved in 100 μL toluene. Matrix and sample were mixed at volume ratio 1:1, and 2 μL of the mixture was applied to the MALDI plate and air-dried.

### Simulation of kinetics

The kinetics of the system were simulated using MATLAB. The corresponding codes are shown in the Supplementary Note 1. The rate constants for the racemization processes were taken from previous work^32,34^.

## Supplementary information

Supplementary Information

## Data Availability

The data shown in the figures can be downloaded from 10.5281/zenodo.3893414
