# Supplementary material for: Amplification of enantiomeric excess by dynamic inversion of enantiomers in deracemization of Au38 clusters
Source: Nat Commun. 2020 Sep 11;11:4562. doi: 10.1038/s41467-020-18357-0 (PMC7486404; doi:10.1038/s41467-020-18357-0)
Supplement: Supplementary file 1 — Supplementary Information [file 41467_2020_18357_MOESM1_ESM.docx]

Supplementary Information

Amplification of Enantiomeric Excess by Dynamic Inversion of Enantiomers in Deracemization of Au_38_ Clusters

Wang et al

SupplementaryInformation

Amplification of Enantiomeric Excess by Dynamic Inversion of Enantiomers in Deracemization of Au_38_ Clusters

Yanan Wang^1^, Belén Nieto-Ortega^1^, Thomas Bürgi^1^*

^1^Department of physical chemistry, University of Geneva, 30 Quai Ernest-Ansermet, 1211 Geneva 4, Switzerland. E-mail: Thomas.Buergi@unige.ch

1. **Supplementary figures and table**


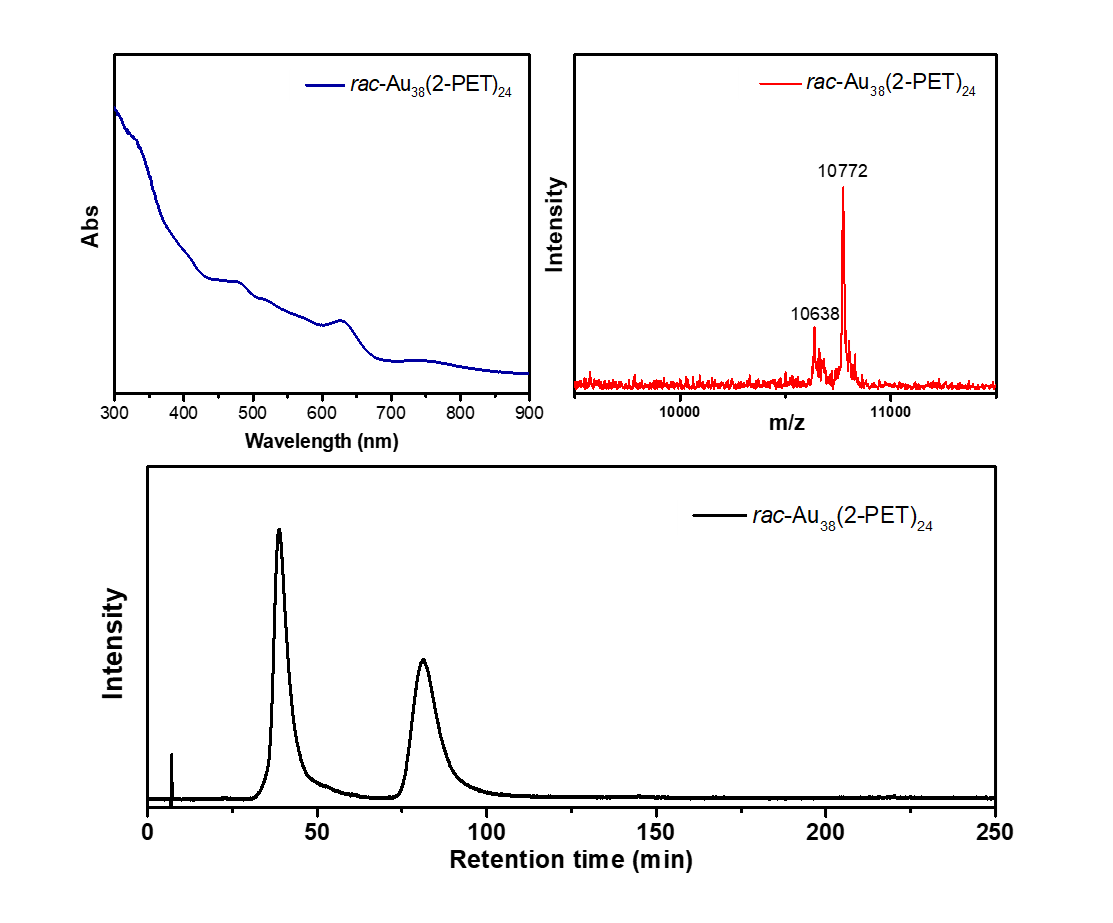


Supplementary Figure 1. Characterization of Au_38_(2-PET)_24_ clusters. Top-left, UV-vis spectrum; top-right, MALDI-TOF spectra. The signal at 10772 corresponds to Au_38_(2-PET)_24_ (calculated mass 10778.4), the signal at 10638 corresponds to Au_38_(2-PET)_23_ (calculated mass 10641.2); bottom, HPLC spectrum.


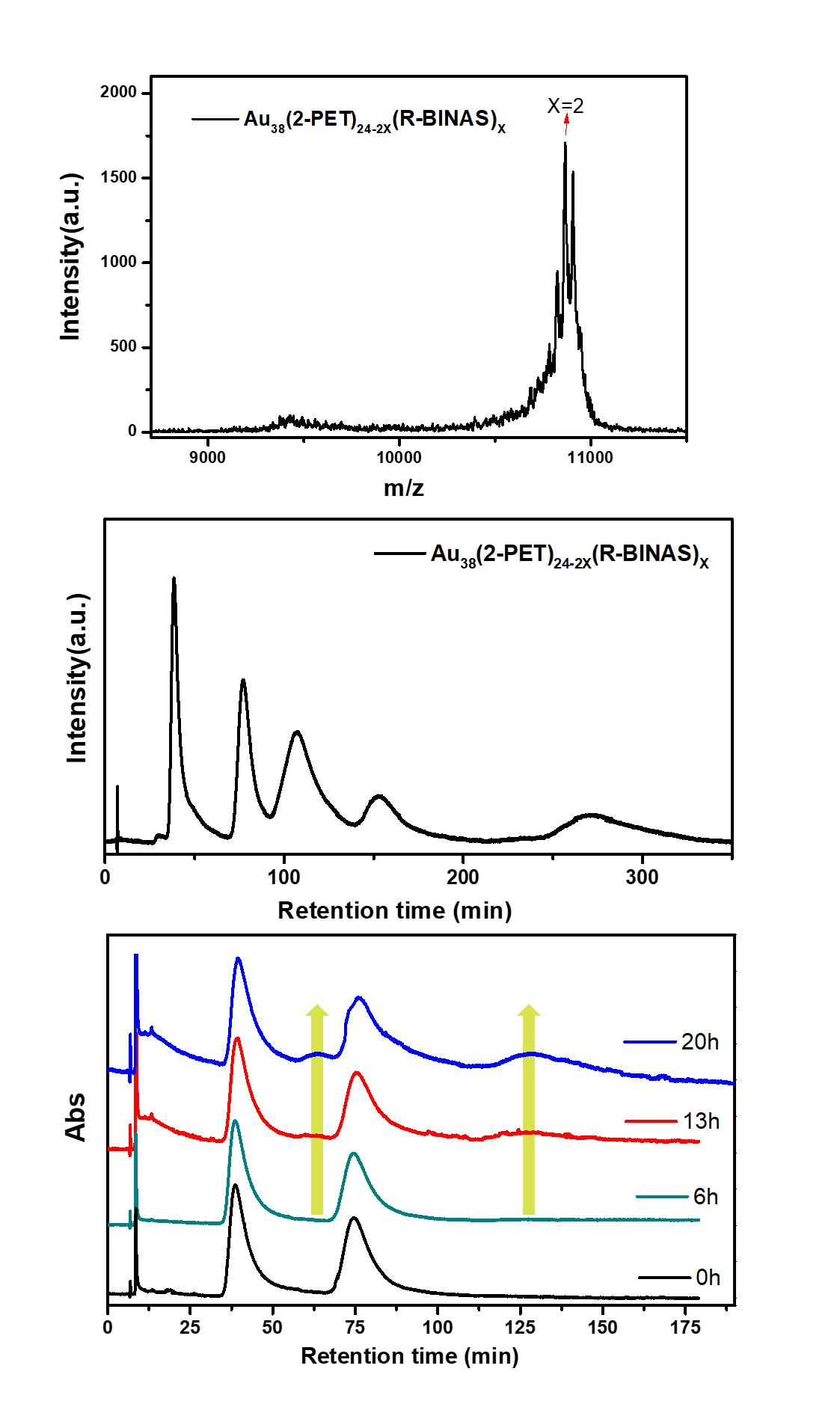


Supplementary Figure 2. Characterization and ligand exchange reaction of Rac-Au_38_ clusters. Top: Characterization of Rac-Au_38_ clusters. MALDI-TOF signal. Middle: HPL chromatogram of Au_38_ clusters after ligand exchange reaction with R-BINAS. Bottom: In-situ HPL chromatograms of Au_38_ clusters during ligand exchange reaction with S-BINAS, arrows indicate the new peaks.


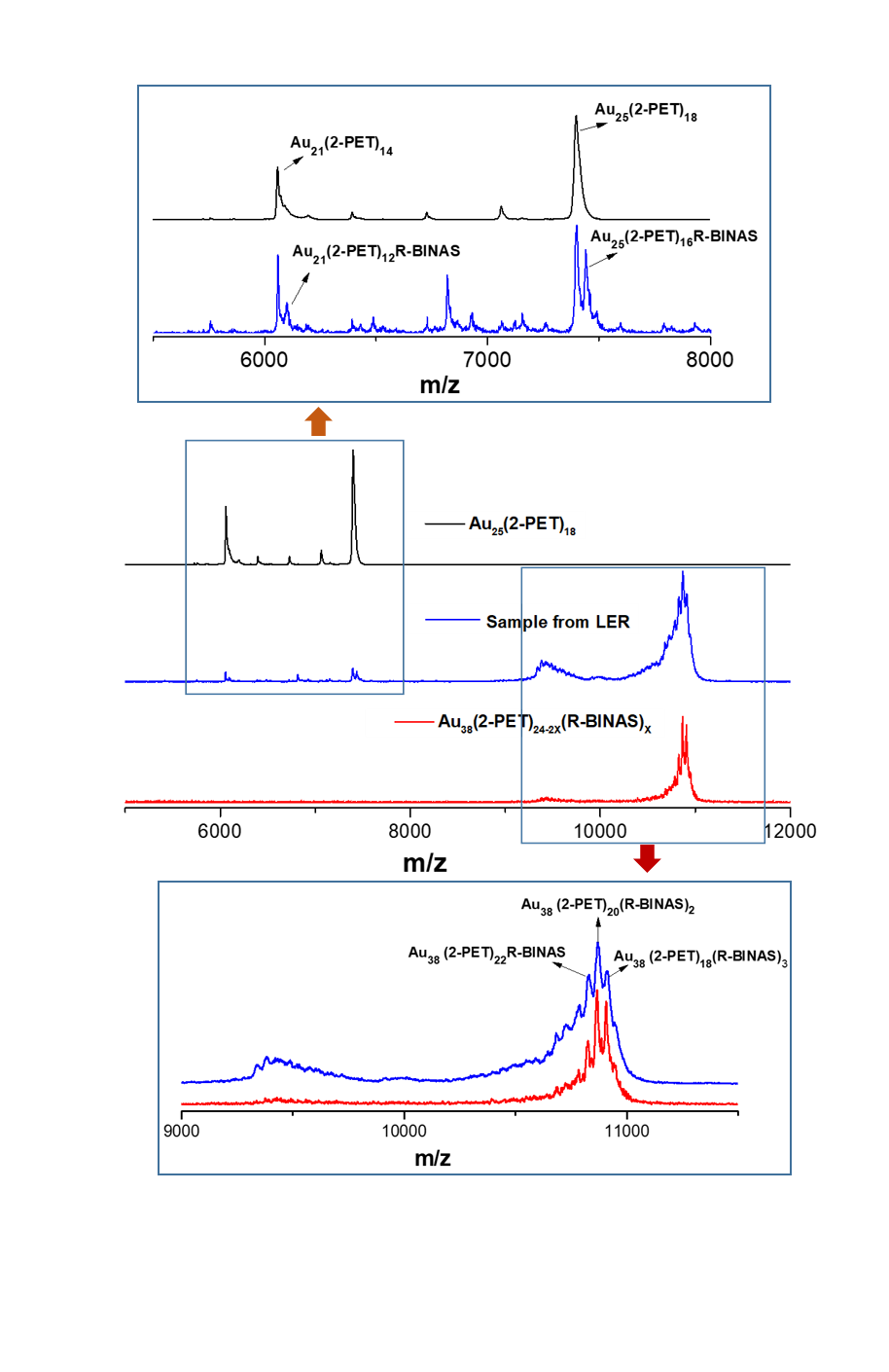


Supplementary Figure 3. MALDI mass spectra of Au_25_(2-PET)_18_ (black), Au_38_(2-PET)_24-2x_(R-BINAS)_x_ (red). After mixing the two samples at 70^o^C for 24h (in the absence of free ligand) the blue MALDI spectra were measured. Top and bottom frames are zoom spectra of specified range.


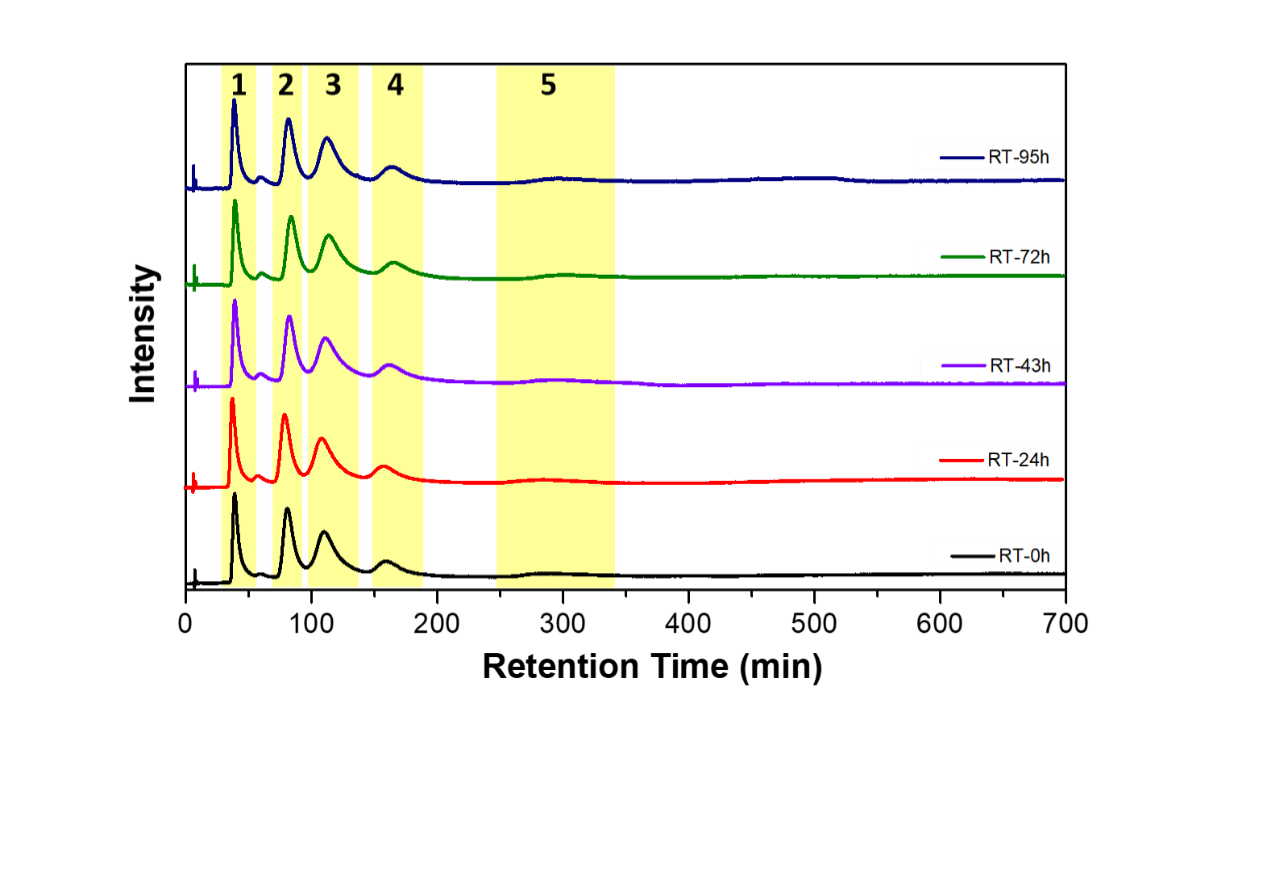


Supplementary Figure 4. HPL chromatograms (0-700min) of a sample containing Au_38_(2-PET)_24_ and R-BINAS-substituted Au_38_-derivatives. The sample was kept at room temperature. The average number of BINAS per cluster was $\bar{x}_{R-BINAS}$ = 0.526.


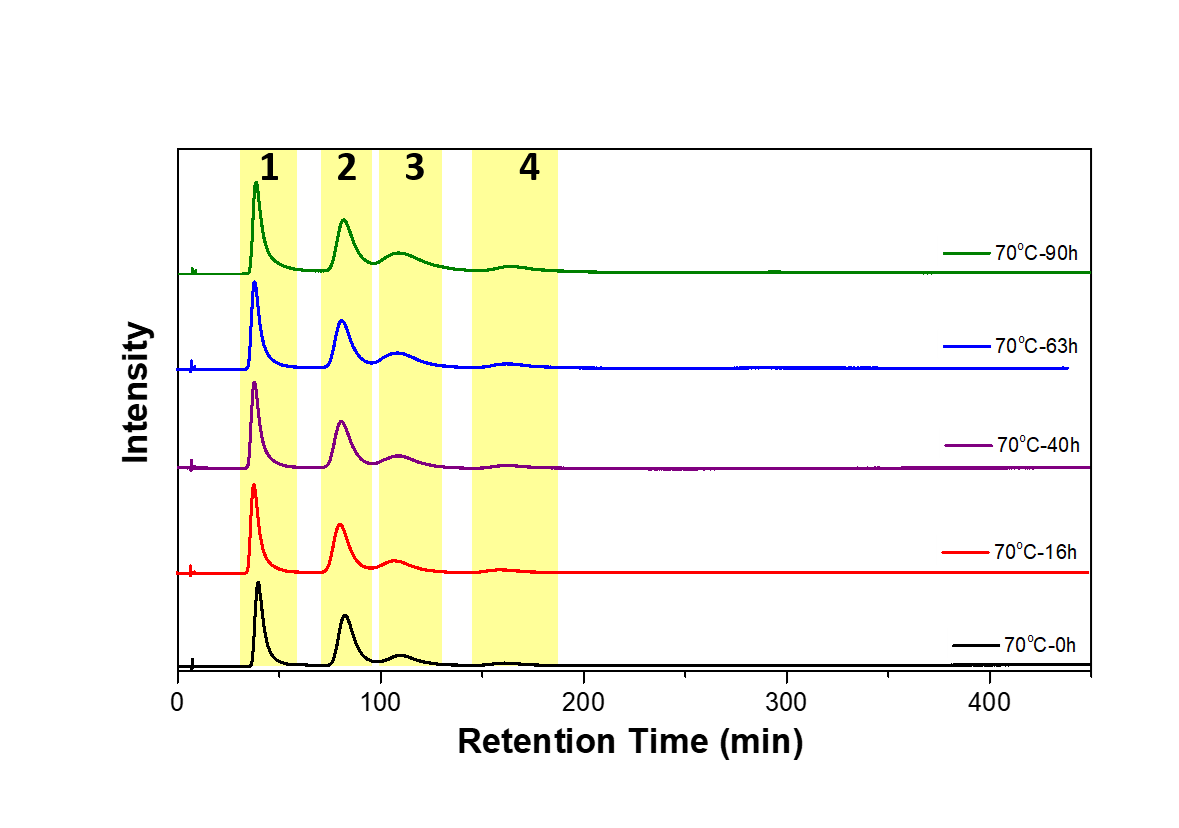


Supplementary Figure 5. HPL chromatograms (0-450min) of a sample containing Au_38_(2-PET)_24_ and R-BINAS-substituted Au_38_-derivatives. The sample was heated to 70^o^C. The average number of BINAS per cluster was $\bar{x}_{R-BINAS}$ = 0.225.


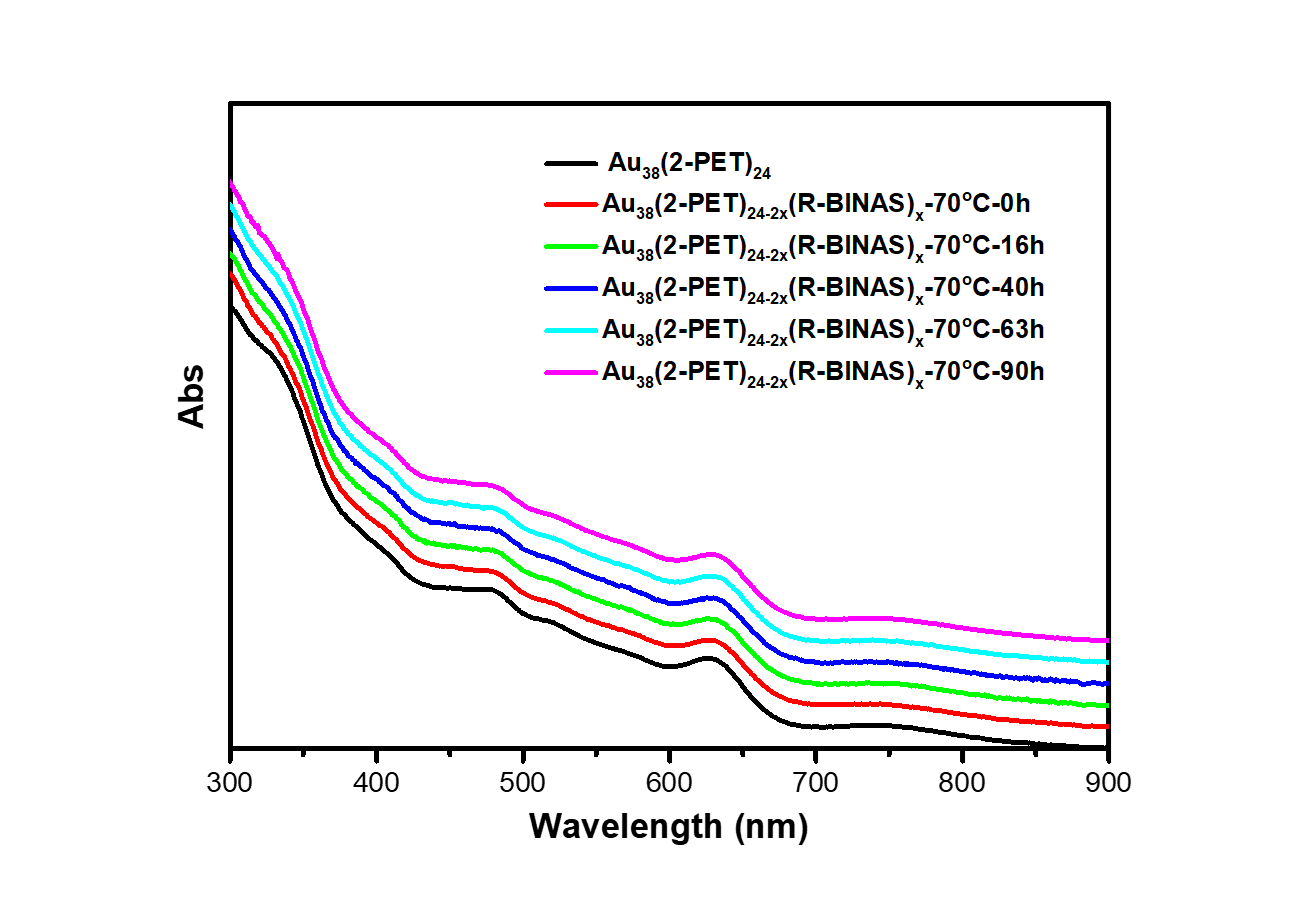


Supplementary Figure 6. UV-vis spectra of Au_38_(SR)_24_ mixture during thermal treatment at different times. The clusters used have an average number of BINAS of $\bar{x}_{R-BINAS}$ = 0.225. Spectra were offset for clarity.


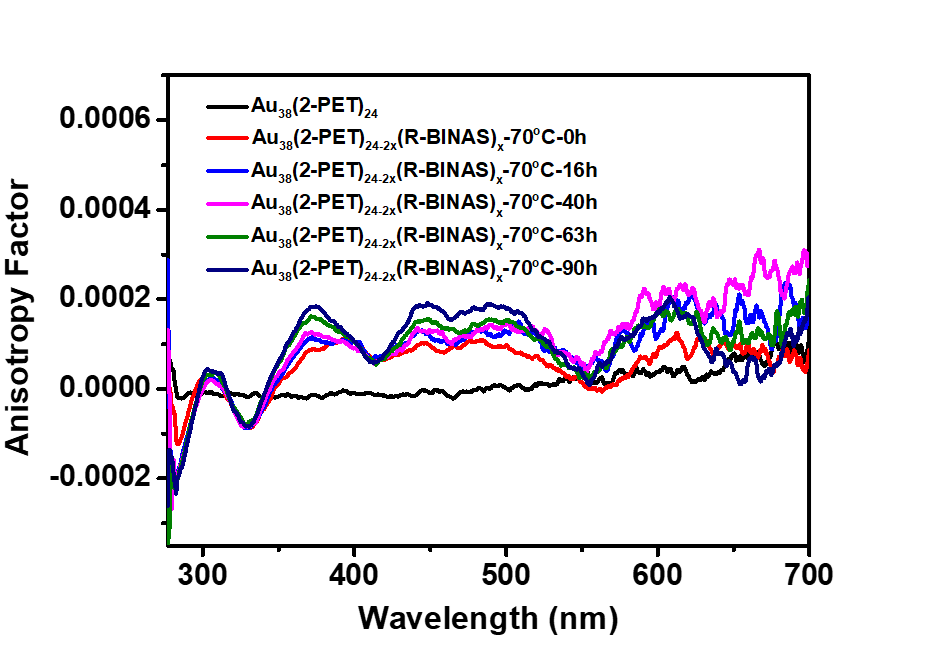


Supplementary Figure 7. Anisotropy factors of Au_38_(SR)_24_ mixture during thermal treatment at different times. The clusters used have an average number of BINAS of $\bar{x}_{R-BINAS}$ = 0.225.


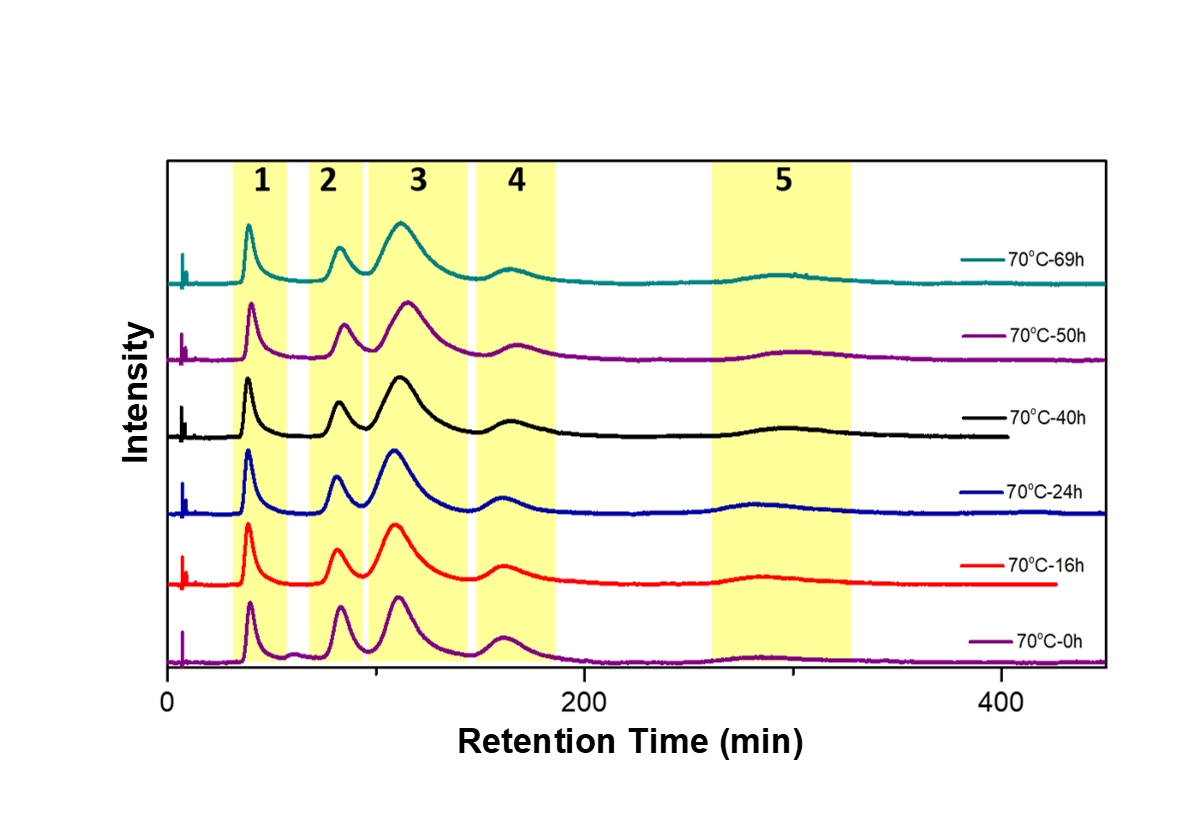


Supplementary Figure 8. HPL chromatograms (0-450min) of a sample containing Au_38_(2-PET)_24_ and R-BINAS-substituted Au_38_-derivatives. The sample was heated to 70^o^C. The average number of BINAS per cluster was $\bar{x}_{R-BINAS}$ = 0.666.


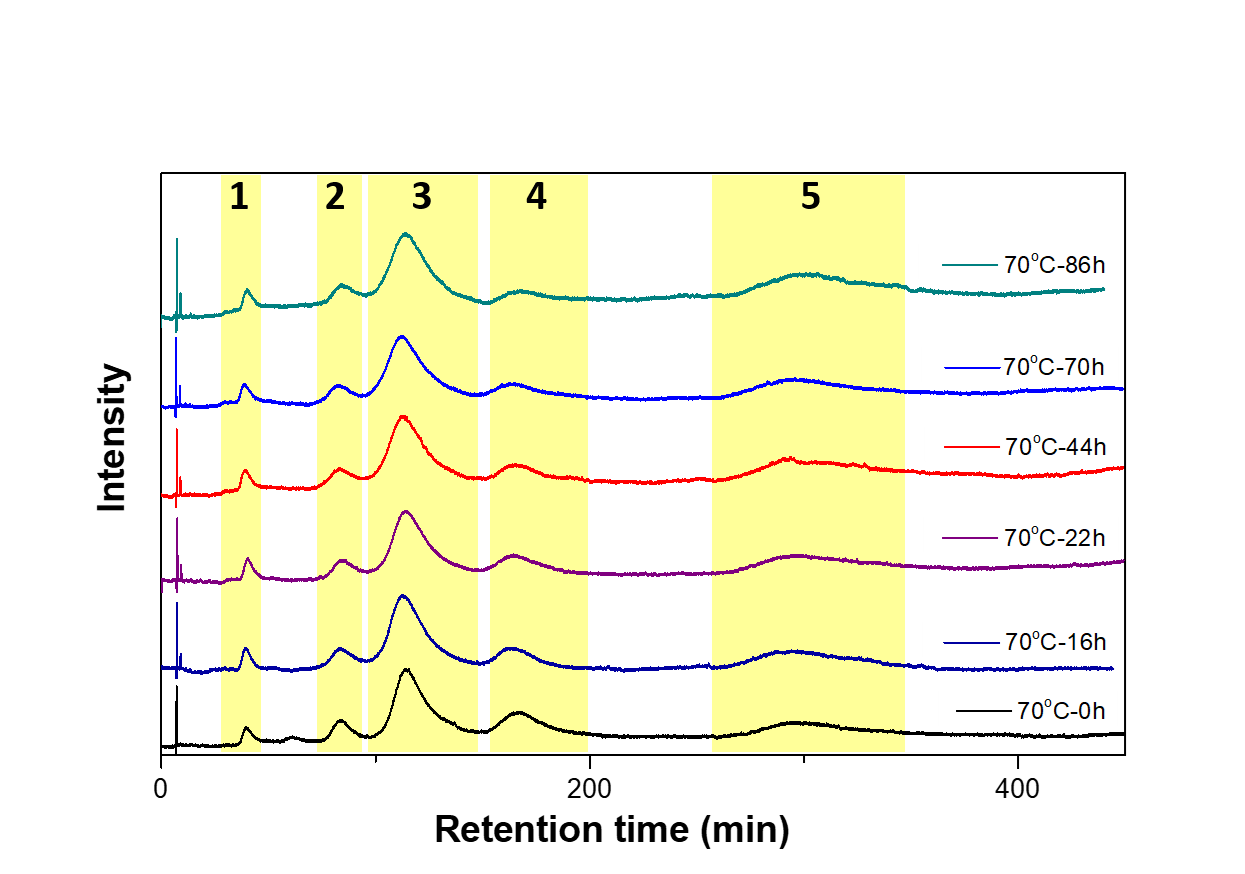


Supplementary Figure 9. HPL chromatograms (0-450min) of a sample containing Au_38_(2-PET)_24_ and R-BINAS-substituted Au_38_-derivatives . The sample was heated to 70^o^C. The average number of BINAS per cluster was $\bar{x}_{R-BINAS}$ = 0.844.

1. **Supplementary Note 1**

Kinetic model and MATLAB routine

$$A-{Au}_{38}\left( 2-PET \right)_{24}\begin{matrix} \underset{\to}{k_{1}} \\ \overset{\leftarrow}{k_{1}} \end{matrix} C-{Au}_{38}\left( 2-PET \right)_{24} \left( \boldsymbol{1} \right)$$

$$A-{Au}_{38}\left( 2-PET \right)_{22}\left( R-BINAS \right)_{1}\begin{matrix} \underset{\to}{k_{2}} \\ \overset{\leftarrow}{k_{3}} \end{matrix} C-{Au}_{38}\left( 2-PET \right)_{22}\left( R-BINAS \right)_{1} \mathbf{(2)}$$

$$A-{Au}_{38}\left( 2-PET \right)_{22}\left( R-BINAS \right)_{1}+C-{Au}_{38}\left( 2-PET \right)_{24}\begin{matrix} \underset{\to}{k_{4}} \\ \overset{\leftarrow}{k_{5}} \end{matrix} C-{Au}_{38}\left( 2-PET \right)_{22}\left( R-BINAS \right)_{1}+A-{Au}_{38}\left( 2-PET \right)_{24} \left( \boldsymbol{3} \right)$$

File 1: ‘derac_mechanism.m’

function dC = derac_mechanism(t,C)

% User friendly variable names.

AR0 = C(1);

CR0 = C(2);

AR1 = C(3);

CR1 = C(4);

% Rate constants.

*k_1_* = 0.000385;

*k_2_* = 0.0000142;

*k_3_* = 0.0000600;

*k_4_* = 0.0000142;

*k_5_* = 0.0000600;

% Rate laws.

r1 = *k_1_**AR0;

r2 = *k_1_**CR0;

r3 = *k_2_**AR1;

r4 = *k_3_**CR1;

r5 = *k_4_**AR1*CR0;

r6 = *k_5_**CR1*AR0;

% Mass balances.

dAR0 = -r1 + r2 + r5 – r6;

dCR0 = r1 - r2 – r5 + r6;

dAR1 = -r3 + r4 - r5 + r6;

dCR1 = r3 - r4 + r5 - r6;

% Assign output variables

dC(1,:) = dAR0;

dC(2,:) = dCR0;

dC(3,:) = dAR1;

dC(4,:) = dCR1;

File 2: ‘derac_runfile.m’

clear all

% Define initial concentrations.

C0 = [1.0, 1.598, 3.376, 1.8];

% Define time span.

tspan = [0, 50000];

% Run ODE solver.

[t, y] = ode15s(@derac_mechanism, tspan, C0);

%Plot.

plot(t, y(:,1),'ro',t ,y(:,2),'bo',t ,y(:,3),'go',t , y(:,4),'ko');

xlabel('time');

ylabel('Concentrations');

legend ({'y(:,1)=AR0','y(:,2)=CR0','y(:,3)=AR1','y(:,4)=CR1'},'Location','southeast');

% prepare for output writing

reswrite = [t,y];

% write to excel file

xlswrite('results.xls', reswrite, 1, 'A1');


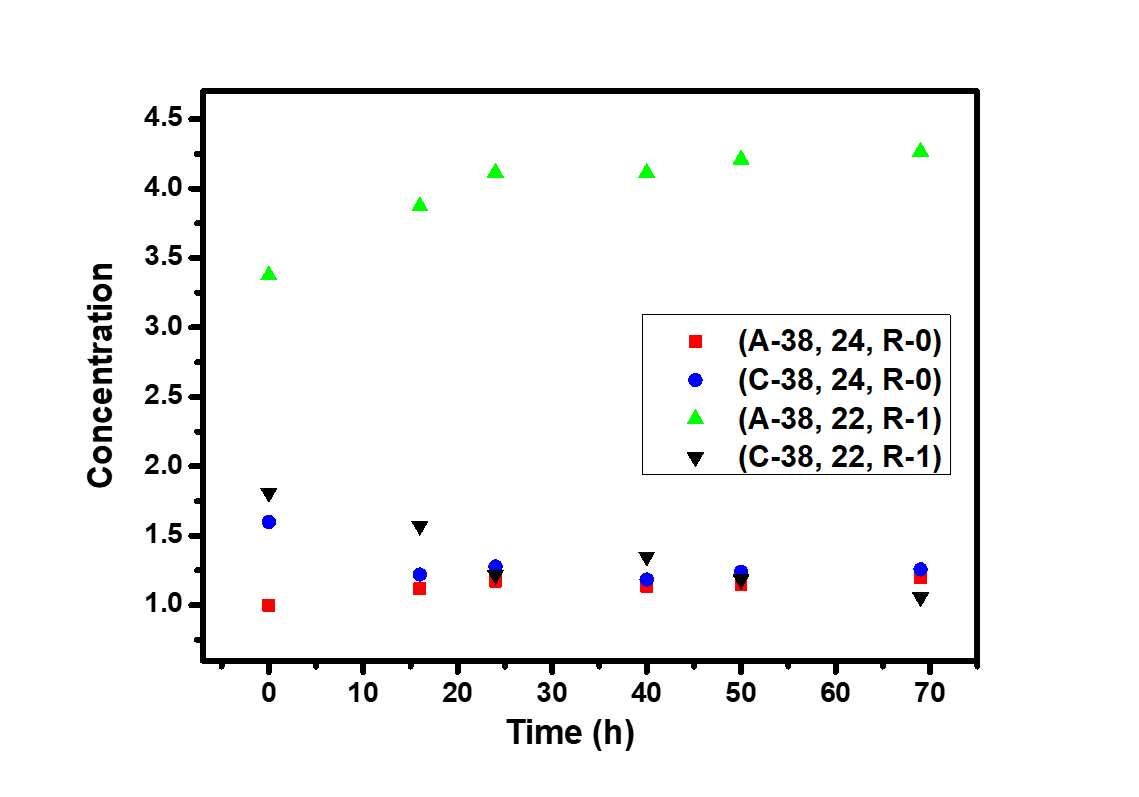


Supplementary Figure 10. Time-dependent concentrations of cluster species. The concentrations are relative to the initial concentration of (A-38, 24, R-0). The sample was heated to 70^o^C. The data extract from Fig S8 ($\bar{x}_{R-BINAS}$ = 0.666).


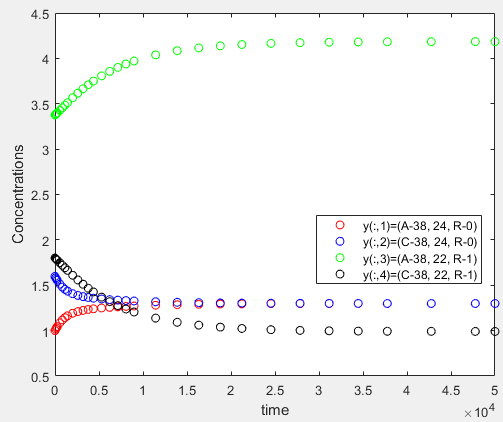


Supplementary Figure 11. Simulated time-dependent concentrations of cluster species using the kinetic model (*k_2_*<*k_3_* and *k_4_*<*k_5_* ). Initial concentrations are the same as the ones for the experiment shown in Supplementary Figure 10.


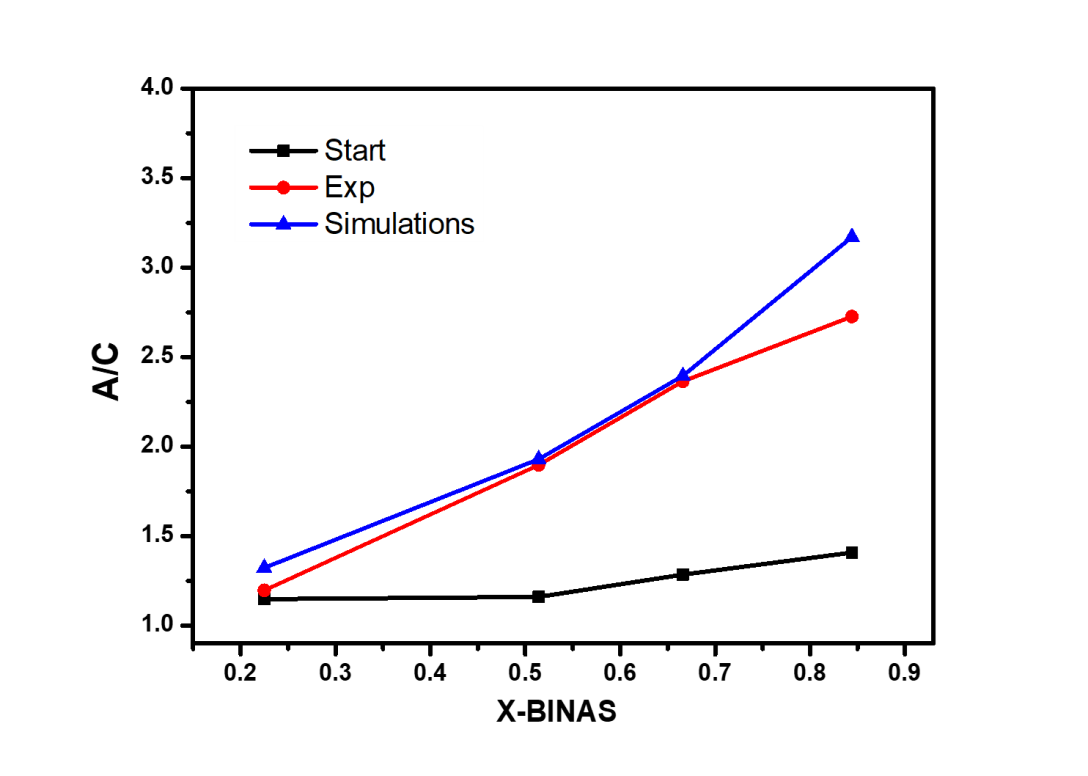


Supplementary Figure 12. Plot of final A/C ratio as a function of $\bar{x}_{R-BINAS}$. Black: experiment, before heating; red: experiment after heating to 70 ^o^C; blue: simulations.


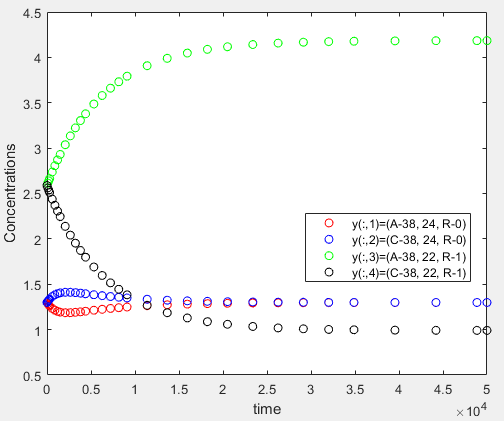


Supplementary Figure 13. Simulated time-depended concentrations of four cluster species. The R-BINAS content of the sample was the same as for the experiment and simulations shown in Figures S10 and S11, respectively, however now starting from a mixture containing equal amounts of clockwise and anti-clockwise clusters.
